# Boron, Manganese, and Zinc Sorption and Leaf Uptake on Citrus Cultivated on a Sandy Soil

**DOI:** 10.3390/plants11050638

**Published:** 2022-02-26

**Authors:** Qudus O. Uthman, Davie M. Kadyampakeni, Peter Nkedi-Kizza, Samuel Kwakye, Neriman Tuba Barlas

**Affiliations:** 1 Soil and Water Sciences Department, University of Florida, 2181 McCarty Hall, Gainesville, FL 32611, USA; q.uthman@ufl.edu (Q.O.U.); kizza@ufl.edu (P.N.-K.); skwakye@ufl.edu (S.K.); 2 Citrus Research and Education Center, University of Florida, 700 Experiment Station Rd., Lake Alfred, FL 33850, USA; 3 Department of Soil Science and Plant Nutrition, Agricultural Faculty, University of Ege, Izmir 35040, Turkey; tuba.barlas@ege.edu.tr

**Keywords:** adsorption, foliar spray, micronutrients, soil application, plant availability

## Abstract

Solute fate in soil-plant continuum could either be soil or leaf uptake or leaching beyond the rooting zone. An adsorption coefficient (K_D_) is an important chemical property to describe the interaction between the solute and soil, affecting the solute movement in soils from one point to another. Boron (B), manganese (Mn), and zinc (Zn) uptake are evident in the leaves as a constituent of photosynthesis and other plant body-building mechanisms for growth and development. This study investigates the availability of micronutrients (B, Mn, and Zn) to citrus trees through modified application methods and rates. Leaf samples were collected from experimental plots arranged in a randomized complete block design, with 4 micronutrient treatments: control, foliar ×1, foliar ×2, and soil ×1. Boron, Mn, and Zn rates were 1.12, 10.08, and 5.60 kg ha^−1^, respectively. Composite soil samples were randomly collected at 5 points, and a 1-point adsorption study was conducted in 4 soil depths at an increment of 15 cm from the soil surface. Adsorption coefficient (K_D_) for Mn and B was 22 and 3 times higher at 0–15 cm than at soil depth of 15–60 cm. The adsorption coefficient (K_D_) for Zn was 2.5 times greater at 0–15 cm than 15–30 cm soil depth, while there was little or no sorption at 30–60 cm. Leaf Mn and Zn concentrations showed that foliar spray was 2 times higher than the soil application method, while B showed that the soil application method was 2 times higher than foliar application method for 2 seasons sampling events. Thus, the behavior of B, Mn, and Zn in the soil via adsorption coefficient (K_D_) reflects the availability of B, Mn, and Zn in the citrus leaves.

## 1. Introduction

Soils are sinks of elemental plant nutrients and sorption is one of the nutrient fates in their environmental cycles [1]. Sorption is a phenomenon (intermolecular interaction, van der Waals forces, ion, ligand exchange, etc.) at the solid-solution boundary whereby solute removed from the aqueous solution cling to the solid surface [2]. Distribution coefficient (K_D_) is an important parameter determining the adsorptive behavior of any soil or porous media for any ion or compounds, which is defined as the ratio of solute concentration in the solid phase to the equilibrium solution after a specified reaction time [3]. Thus, knowledge of K_D_ is a key factor affecting the mobility and retention of essential nutrients in soils.

Micronutrients are key elements that plants require in minute quantity for their growth does not imply that their role is minor, but their absence can cause a severe reduction in yield [4]. They play vital roles in metabolic and cellular processes and functions, but the level of micronutrients required by plants differs from one specie to another [5]. They are protein constituents and are redox-active ions, which is the foundation for their occurrence as catalytically active cofactors in enzymes [5]. Citrus is sensitive to deficiencies of boron [B], copper [Cu], iron [Fe], manganese [Mn], zinc [Zn], and molybdenum [Mo] [6,7]. The quantity of micronutrients in most soils is not a good sign of its availability to plants [8]. 

Citrus micronutrient deficiencies have been seen in uncultivated and shallow soils with a high-water table, extremely sandy areas, and calcareous soils [9]. Soil and foliar application of micronutrients have been recommended to correct deficiencies in citrus [7]. The management of essential nutrients as a means of alleviating tree growth, fruit yield and its quality in citrus groves is not well understood to provide conclusive nutrient management recommendations to growers, and thus deserves additional research [10]. The aim of this study was to (1) determine the adsorption, distribution, and availability of micronutrients in sandy soil; (2) compare the effectiveness of foliar and soil application method of B, Mn, and Zn on citrus trees; (3) compare foliar application rates of B, Mn, and Zn for citrus trees.

## 2. Materials and Methodology

### 2.1. Description of the Study Site

This study was performed on ‘Valencia’ sweet orange [*Citrus sinensis* (L.) Osb.] trees on Citrumelo Swingle rootstock (*Citrus paradisi* Macf. × *Poncirus trifoliata* (L.) Raf.) spaced at 1.8 m by 4.6 m at the Citrus Research and Education Center in Lake Alfred, FL (28.09° N, 81.75° W) which was planted in 2012. The soil of the study site is Candler fine sand, classified as Hyperthermic, coated Lamellic Quartzipsamments formed from eolian or sandy marine deposits [11]. Soil bulk density was determined using the core method (Table 1, [12]). Particle density was determined using the pycnometer method (Table 1, [12]). The soil pH is approximately 6 and organic matter content is less than 0.5% [13,14]. Lowering the pH specifically for this micronutrient’s availability would impair the availability of essentially important macronutrients. Soil moisture was monitored, and data were collected at 4 depths from the soil surface to 60-cm at equal intervals using 10-Hscapacitance sensors (Meter Group, Pulman, WA, USA) [14]. The trees were supplied with 2240 kg ha^−1^ fertilizer blend at the University of Florida Institute of Food and Agricultural Sciences (UF/IFAS) recommendation rate containing a known amount of nutrients in 4 splits per year given in percentage as: 9.75% N, 2% P_2_O_5_, 13% K_2_O, 2.28% Ca, 2.5% Mg, 11.69% S, 0.03% B, 0.27% Fe, 0.55% Mn and 0.19% Zn.

### 2.2. Experimental Design and Treatment Application

Treatment plots contained ten trees where the middle eight trees were used for measurements. There were 9 rows with each row sub-divided into 4 plots receiving B+Mn+Zn applications in 3 splits per year as follows: (1) standard soil B+Mn+Zn applied (control, 1× = 1.12 kg B ha^−1^; 10.08 kg Mn ha^−1^; 5.60 kg Zn ha^−1^), (2) standard soil B+Mn+Zn applied + foliar applied B+Mn+Zn based at 1× UF/IFAS recommendations (Morgan and Kadyampakeni, 2020), (3) 2× foliar applied B+Mn+Zn at UF/IFAS recommendations+ standard soil B+Mn+Zn application, and (4) 2× soil applied UF/IFAS recommendations (2× = 2.24 kg B ha^−1^; 20.16 kg Mn ha^−1^; 11.20 kg Zn ha^−1^). The soil treatment was applied within 45 cm of the tree radius, while the foliar treatment was applied as foliar sprays. All of the trees were irrigated by 40 L h^−1^ microsprinkler emitters (one emitter per two trees) to meet the daily evapotranspiration demand with a wetting radius of 1.5 m, at 8 a.m. and 1 p.m. daily. 

### 2.3. Adsorption Study

One-point sorption isotherms were determined to estimate the sorption coefficient of B, Mn, and Zn as a function of soil depth, similar to the method of Uthman et al. [13,14]. The initial concentration of 2, 15, and 10 mg L^−1^ for B, Mn, and Zn were used based on UF/IFAS recommended rate, assuming a 2-cm depth of incorporation into the soil. The soil: solution ratio used was 1:2, shaken for 24 h, centrifuged at 10,000 rpm for 20 min, filtered, and analyzed for B, Mn, and Zn using ICP—Atomic Emission Spectrometry (ICP-AES). The amount of B, Mn, and Zn adsorbed to the soil was calculated from the difference between the initial and equilibrium solution concentrations as follows:

(1) $$S_{e}=\frac{v}{m}\left(C_{0}-C_{e}\right)$$

where *S_e_* is the adsorbed concentration at equilibrium (mg kg^−1^), *v* is the initial volume of solution (L), *m* is the soil mass (kg), *C*_0_ is the initial concentration of the standard solution (mg L^−1^), and *C_e_* is the soil solution concentration at equilibrium (mg L^−1^). The linear sorption isotherm was determined from the model below:

(2) $$S_{e}=K_{D}C_{e}$$

where *K_D_* is the sorption coefficient (L kg^−1^).

### 2.4. Soil and Leaf Sampling and Analysis

Soil and leaf samples were collected and analyzed for B, Mn, and Zn before and after treatments application, similar to Uthman et al. [13,14]. Soil samples were collected in June 2018, February 2019 and September 2019 and leaf samples were collected in October 2017, June 2018, November 2018, February 2019, September 2019, and February 2020 for the study period. Soil samples were collected at 4 depths from the soil surface to 60-cm at equal intervals. Soil samples were dried at 100 °C for 24 h and analyzed for B, Mn, and Zn using Mehlich III extraction procedure with 1:10 soil to solution ratio (mass: volume) and determined using inductively coupled plasma-mass spectrometry [13,14]. Leaf samples were processed and analyzed using acid digestion method to determine B, Mn, and Zn inductively using coupled plasma–optical emission spectrometry [13,14].

### 2.5. Statistical Analysis

For soil analysis, micronutrient treatments (four levels) were used as fixed factors, and soil depth (four levels) were used as random factors. A linear mixed effect model using linear mixed effect (lmerTest) package by Kuznetsova et al. [15] was used to analyze the variance between treatments effect, and Tukey honest significant difference (HSD) was used to separate means using a significance level of 0.05. For leaf analysis, analysis of variance was conducted with R statistical packages using micronutrient treatments as fixed factor treatments in a randomized complete block design. The R software interface was used for this analysis. 

## 3. Result and Discussion

### 3.1. Adsorption Study in a Sandy Soil

**Figure 1:**
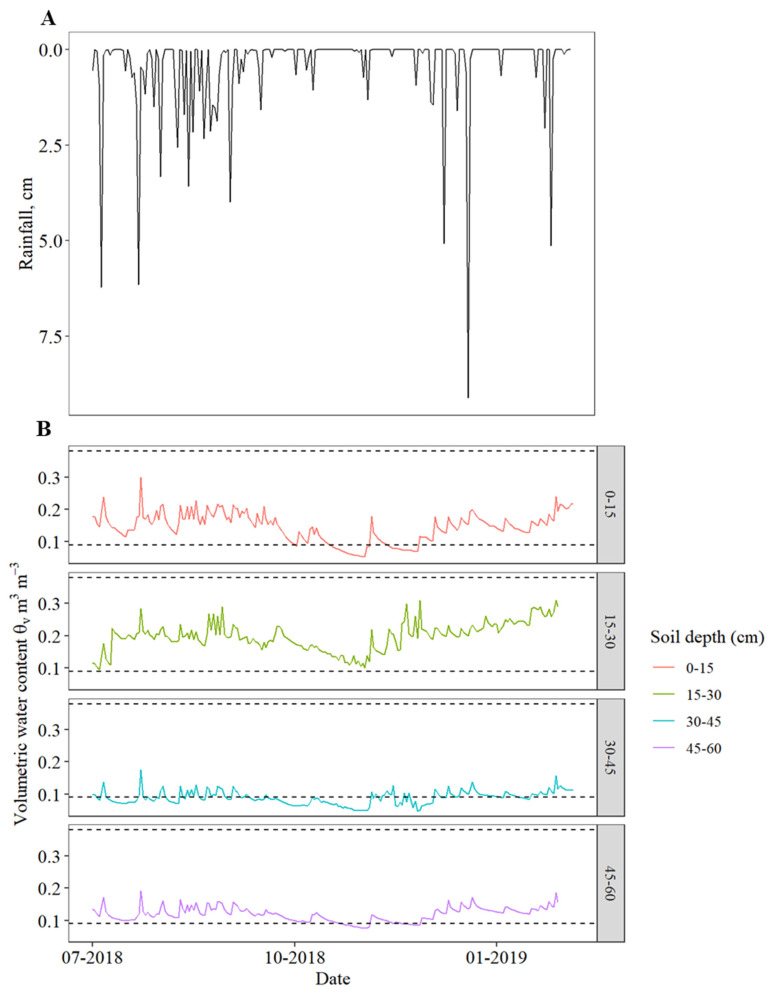
(**A**)—Rainfall from 1 July 2018–5 February 2019; (**B**)—Soil moisture distribution in the 0–60 cm depth at an increment of 15 cm, from 1 July 2018–5 February 2019. The upper and lower dash lines in each depth are water content at saturation and field capacity.

Boron showed negligible sorption throughout the soil depth of 0–60 cm (Table 2; [13]). The adsorption study revealed high Mn and Zn sorption coefficients at 0–15 cm soil depth for Mn and 0–15 and 15–30 cm soil depths for Zn, while 15–60 and 30–60 cm for Mn and Zn showed negligible sorption (Table 2). Manganese sorption coefficients at 0–15 cm were 22 times greater than the soil depth of 15–60 cm, while Zn sorption coefficients at 0–15 cm and 15–30 cm depth were, respectively, 130 and 50 times greater than 30–45 cm and 45–60 cm soil depths (Table 2). Assuming that during 1 of the heaviest rainfall events (for instance summer rainfall events in Florida), the soil water content in the 0–15 cm depth would be close to saturation (Table 1 [θ_s_ = 0.41]) and the retardation factor^®^ of Mn and Zn would be 9 and 25, respectively, (Table 2). This implies that even if water moved down to 50 cm from the soil surface, Mn and Zn would have moved only 9 cm and 2 cm into the soil profile. Mn and Zn never moved below the 30 cm and 15 cm depth, respectively. Since the control and soil treatments have nearly equal concentrations in June 2018 and February 2019 but showed high Mn and Zn concentrations in September 2019 (Table 3 and Table 4). It is also clear that water content during this study was close to field capacity below the 30 cm depth, implying negligible downward water movement from above 30 cm depth (Figure 1B). Thus, strong adsorption retained Mn and Zn in the 0–15 cm soil layer. Meanwhile, we observed the movement of B over relatively deeper soil depth by mass flow and diffusion, similar to Scott et al. [16]. However, this was possible by replacing water lost through evapotranspiration with irrigation which kept water available throughout the whole experimental study.

Maintaining soil moisture content at field capacity could be an important factor affecting B, Mn, and Zn plant availability. At the field capacity demonstrated (Figure 1B), it is implied that the potential gradient was one and water movement in the soil profile equals unsaturated hydraulic conductivity in the soil [that is, at field capacity, water flux (q) = K(θ_FC_)]. Using Mualem model [17], pore water velocity is estimated be about 1.5 cm day^−1^. Thus, water movement at field capacity is very slow. It means transpiration demand of the tree is pulling water (plus nutrients) from the soil, but Mn and Zn, distinct from B, were moving with water in a limited amount such that the leaves could show deficiency symptoms.

Hippler et al. [18] showed that Mn and Zn soil application could supply sweet orange trees with nutrient requirements, but low fertilizer solubility and high soil sorption capacity limiting fertilization efficiency was demonstrated in this study. Distinct from B, most of Mn and Zn applied were adsorbed to the 0–15 cm and 0–30 cm soil depth matrix, the soil solution concentration would be very low to supply the roots via mass flow [19]. Due to low Mn and Zn concentration in the soil solution, supplying the roots with mass flow accounts for only a small fraction of plant demand [19]. The soil was predominantly sandy with a low percentage of organic matter (<1%), but the soil clay size fraction could have been responsible for Mn and Zn sorption in the 0–15 and 15–30 cm soil depth. The epipedon of the soil under study has sand coatings that were systematically high in hydroxy-interlayered minerals and unremarkably gibbsite [20]. Manganese and Zn might have formed stable complexes with clay minerals and high molecular-weight organic compounds that exist as insoluble complexes and might have made Mn and Zn applied to the soil unavailable for uptake in the short-term but could be released over time [21].

### 3.2. Soil Micronutrients Distribution in a Sandy Soil

Boron showed no changes in soil concentration as a function of depth throughout the sampling events (Table 3 and Table 4). Boron concentration in the summer (June 2018) was 1.33 times greater than winter (February 2019) and Fall (September 2019). The 0–15 cm soil depth showed 3 times higher Mn concentration than the 15–60 cm soil depths in summer (June 2018), winter (February 2019), and fall (September 2019) for both treatments and control plots (Table 3 and Table 4). Manganese concentration for both treatment and control plots in the fall was 2 times more than summer and winter (Table 3 and Table 4). The 0–15 cm soil depth showed 3- and 6-times higher Zn concentration than the 15–30 cm and 30–60 cm soil depths, respectively, in summer sampling events for both treatment and control plots (Table 3 and Table 4). The 0–15 cm soil depth also showed 2- and 5-times greater Zn concentration than the 15–30 cm and 30–60 cm soil depth, respectively, in winter sampling events for the treatment and control plots (Table 3 and Table 4). The 0–15 cm soil depth also showed 2- and 4-times greater Zn concentration than the 15–45 cm and 45–60 cm soil depths, respectively, in fall sampling events for the treatment and control plots (Table 3 and Table 4). The 45–60 cm soil depth showed 2 times less Zn concentration than the 15–45 cm in fall sampling events for the treatment and control plots (Table 3 and Table 4). Zinc concentration for the 15–30 cm soil depth in winter was 1.5 times more than the summer sampling event (Table 3 and Table 4). The amount of B, Mn, and Zn applied plus the background fertilizer blend was lower than the concentration in the soil for the control plots in the 0–15 cm layer at all the sampling events (Table 3 and Table 4). This implies that Mn and Zn were probably in the soil in forms that were not readily available to the citrus roots while B was available to the citrus roots.

Approximately 8 kg Mn ha^−1^ and 3 kg Zn ha^−1^ (Mn and Zn treatment plus background fertilizer blend) were applied in the first split, and 74 kg Mn ha^−1^ and 34 kg Zn ha^−1^ were present in the soil at the June 2018 sampling event in the 0–15 cm soil depth. A total of 20 kg Mn ha^−1^ and 10 kg Zn ha^−1^ was applied in 3 splits (Mn and Zn treatment plus background fertilizer blend) 59 kg Mn ha^−1^ and 37 kg Zn ha^−1^ were present in the soil at the February 2019 sampling event in 0–15 cm soil depth. Moreover, 2 times more than what was found present in 0–15 cm soil depth in February 2019 was found present in September 2019 for Mn and Zn. The rainfall from the first treatment application to sampling was approximately 47 cm with a peak of 25 cm in May 2018 compared to the second treatment application to the time of sampling, which was about 108 cm with a double peak of 22 cm and 27 cm in July and August 2018, respectively (Figure 1A). The rainfall pattern for the year 2018 was also similar for the years 2019 and 2020. High Mn and Zn concentrations at the upper depth proved strongly adsorbed and retarded in the soil, even if there was rainfall and irrigation supplied to meet the crop evapotranspiration. Thus, Mn and Zn were present in high concentrations in the soil, more than what had been applied. This might also be ascribed to the fact that this soil has sand coatings with iron and aluminum sesquioxide which might bind Mn and Zn [21].

Boron has a consistent and narrow range of soil concentrations throughout the whole sampling event. This means that, irrespective of the season of soil sampling events, there is enough B in the soil to supply the roots and not much affected by temperature to hinder its availability to the plants [13]. Manganese and Zn deficiencies in Huanglongbing (HLB)-affected trees are more pronounced during the cool, wet seasons and often disappear in a warmer season. Comparing the season of soil samples analysis, the fall months showed Mn and Zn concentrations in the soil to be significantly higher than the cool winter and warmer summer months. The low concentration of Mn and Zn in the soil during these seasons could be caused by the slow diffusion process responsible for the transport of Mn and Zn to the plant roots [21]. Soil temperature, which could be affected by season, could increase/decrease Mn and Zn availability through its solubility and diffusion [21].

The relationship between elemental nutrients in the soil is vital to plant’s nutrient management. A greenhouse study of 2-year-old ‘Valencia’ (*Citrus sinensis* (L.) Osbeck) citrus trees on variable fertilizer rate study showed that soil Mn negatively correlated with B and Zn (Table 5). This helps in our understanding that when devising a fertilizer recommendation, only optimal rates for each fertilizer should be applied. This negative correlation could be of a concern if one nutrient is over applied, creating an antagonistic effect among the nutrients.

### 3.3. Seasonal Leaf Response to Micronutrients 

**Figure 2:**
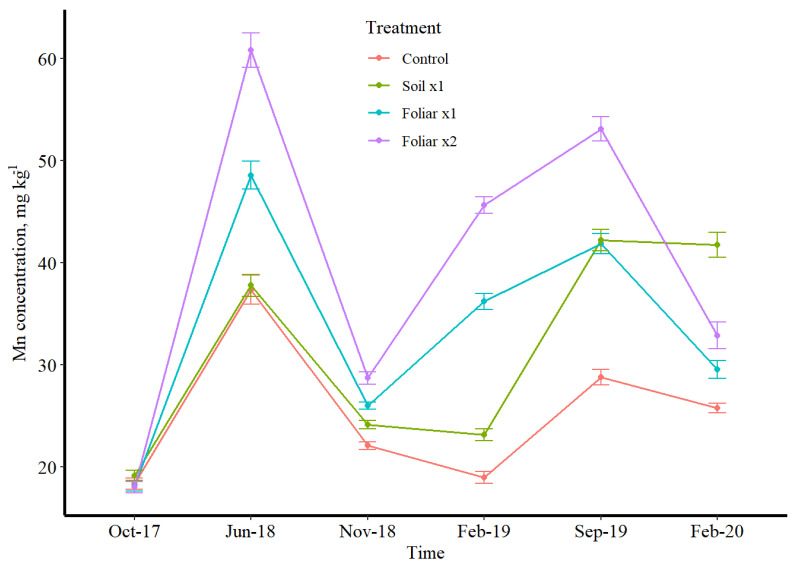
Mn leaf concentration over time in different micronutrient treatments (control, soil ×1. foliar ×1, and foliar ×2). B, Mn, and Zn rates were 1.12, 10.08, and 5.60 kg ha^−1^. Lines represent data with 95% confidence intervals.

**Figure 3:**
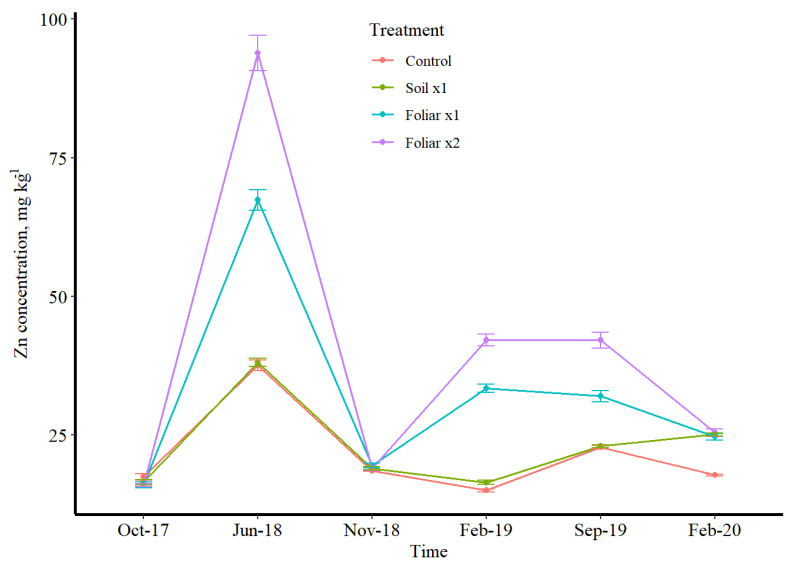
Zn leaf concentration over time in different micronutrient treatments (control, soil ×1. foliar ×1, and foliar ×2). B, Mn, and Zn rates were 1.12, 10.08, and 5.60 kg ha^−1^. Lines represent data with 95% confidence intervals.

**Figure 4:**
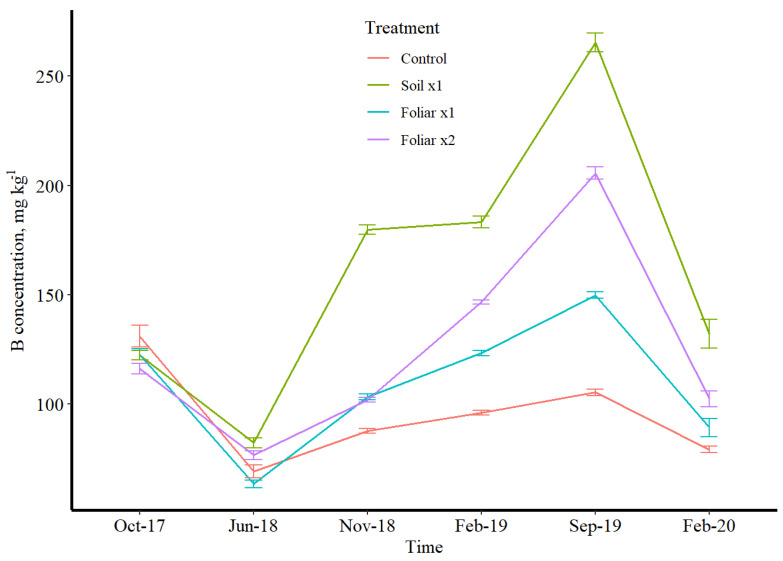
B leaf concentration over time in different micronutrient treatments (control, soil ×1. foliar ×1, and foliar ×2). B, Mn, and Zn rates were 1.12, 10.08, and 5.60 kg ha^−1^. Lines represent data with 95% confidence intervals.

Manganese and Zn followed the same variation pattern concerning treatments, while B was the opposite (Figure 2, Figure 3 and Figure 4). Boron has a depressed concentration, while Mn and Zn had an elevated concentration in June 2018 (Figure 2, Figure 3 and Figure 4). Manganese and Zn concentration showed that foliar spray was 2 times higher than soil application, while B showed that soil application was 2 times higher than foliar application for mostly winter and fall sampling time (Figure 2, Figure 3 and Figure 4). The double rates of foliar B, Mn and Zn treatments showed/resulted in a higher leaf concentration than a single foliar treatment rate (Figure 2, Figure 3 and Figure 4). Conversely, a greenhouse study by Kwakye et al. (accepted paper for publication) found a linear response of leaf Mn concentration to soil Mn application, where the higher rates accumulated more than the lower rates in the leaf.

Boron has shown to be enough with or without fertilizer application since the soil treatment has more than enough when compared to the foliar treatments. Irrespective of the treatments, B has proven to be sufficient in the soil and is far beyond the optimal level recommended by UF/IFAS nutrient management guidelines for HLB-affected trees [22]. Manganese and Zn absorption is rapid following foliar application, with a sharp decrease in the absorption rate after a few hours [23]. At least 50% of Mn and Zn applied were absorbed within 24 h of application [24]. Adsorptive characteristics of the leaf cuticle described by Orgell [25] as a semi-lipoidal cation exchange membrane are similar to the selective permeable amphoteric membrane [26], which might have affected intra-cuticular penetration. The relative adsorptive capacity of an isolated cuticle for acidic substances decreases and increases for basic substances [25]. This shows a striking similarity to the effect of pH of the applied solution on the relative rates of absorption [25]. The double rate of the foliar application method of Mn and Zn might increase acidity, which increased its intra-cuticular penetration. There is almost a universal agreement that foliar absorption rates for Mn and Zn are more significant for young leaves than old ones [27] since the latter are sampled after 4 to 6 months. The primary mechanism for Mn and Zn absorption is diffusion since it is absorbed and rapidly moves throughout the plant [24]. Wallihan and Heymann-Herschberg [28] showed that Zn sulfate and sulfur sprays or dusts might be combined to control citrus rust mite and Zn deficiency in sweet orange trees. Gomes et al.’s [29] study has shown that solubilities of Mn and Zn are the main factor responsible for faster absorption rates through the leaves, and sulfate salt of these nutrients could have resulted in better utilization efficiency as observed in this study as the source of fertilizer used for this study was sulfate salts dissolved in water for foliar spray.

## 4. Conclusions

This research confirmed preliminary, short-term observations [13,14] and showed that soil-applied nutrients could be strongly adsorbed to the upper 0–30 cm soil depth for Mn and Zn; the foliar application method of Mn and Zn at the single recommended rate for Mn and double recommended rate Zn proved to be effective to meet plants micronutrients demand. Distinct from Mn and Zn, B does not adsorb on sandy soils and could leach easily. The negative correlation of Mn with B and Zn and the positive correlation of Mn with Fe and Cu improves our understanding that when devising a fertilizer recommendation, only optimal rates for each fertilizer should be applied. This negative correlation could be of a concern if one nutrient is applied at higher-than-normal doses, creating an antagonistic effect among the nutrients. Applying B to meet micronutrient demand based on effective economic cost would be good because the soil and foliar applied B at a single or double rate resulted in optimum leaf critical concentration. However, keeping soil moisture content at field capacity would reduce leaching of B, Mn, and Zn beyond the tree rooting zone. 

## Figures and Tables

**Table 1:** Soil bulk density, particle density, volumetric moisture content at saturation and field capacity across four soil depths from the soil surface.

Soil Depth	ρ_s_	ρ_b_	θ_s_	θ_FC_
	g cm^−3^	g cm^−3^	cm^3^ cm^−3^	cm^3^ cm^−3^
0–15	2.65	1.56	0.41	0.07
15–30	2.65	1.67	0.37	0.10
30–45	2.65	1.61	0.39	0.10
45–60	2.65	1.63	0.38	0.09

ρ_s_ = Particle density; ρ_b_ = Bulk density. θ_s_ = soil moisture at saturation; θ_FC_ = soil moisture at field capacity.

**Table 2:** Adsorption coefficient, retardation factor at soil saturation and field capacity across four soil depths from the soil surface.

Soil Depth	K_D_, cm^3^ g^−1^			R (θ_s_)			R (θ_FC_)		
	B	Mn	Zn	B	Mn	Zn	B	Mn	Zn
0–15	0.10	2.20	6.47	1.38	9.34	25.54	3.23	50.03	145.19
15–30	0.03	0.13	2.68	1.14	1.59	13.12	1.50	3.17	45.81
30–45	0.04	0.10	0.05	1.16	1.41	1.21	1.64	2.61	1.81
45–60	0.03	0.09	0.05	1.13	1.38	1.21	1.54	2.63	1.91

R = Retardation factor; K_D_ = adsorption coefficient.

**Table 3:** Factor effects and their significance level for B, Mn, and Zn.

Source of Variation	Effect	B	Mn	Zn
Treatment	FE	**	***	***
Time	FE	***	***	***
Treatment × Time	FE	***	***	***
Depth	RE	*	***	***
Rep × Depth	RE	ns	***	***

Significant at * *p* < 0.05, ** *p* < 0.01, and *** <0.001. ns not significant. FE Fixed effect; RE Random effect.

**Table 4:** Soil B, Mn, and Zn distribution as a function of soil depth and sampling time with 95% confidence intervals (as measured in Control (standard rate) plots and soil applied (Treatment 4 plots)).

	B		Mn		Zn	
Soil Depth, cm	Control	Soil ^1^	Control	Soil	Control	Soil
June 2018	^__________________________________^mg kg^−1______________________________________^					
0–15	0.4 ± 0.004	0.5 ± 0.005	49.9 ± 0.7	72.6 ± 1.2	24.7 ± 0.5	35.7 ± 0.7
15–30	0.3 ± 0.002	0.4 ± 0.002	15.2 ± 0.4	19.4 ± 0.4	10.5 ± 0.6	8.7 ± 0.3
30–45	0.3 ± 0.002	0.4 ± 0.002	8.5 ± 0.2	11.5 ± 0.3	5.1 ± 0.2	4.7 ± 0.1
45–60	0.3 ± 0.005	0.4 ± 0.002	8.2 ± 0.2	10.1 ± 0.2	5.6 ± 0.2	4.8 ± 0.1
February 2019						
0–15	0.3 ± 0.002	0.4 ± 0.004	45.4 ± 0.6	55.3 ± 0.8	28.8 ± 0.5	31.6 ± 0.6
15–30	0.3 ± 0.003	0.3 ± 0.003	15.4 ± 0.3	17.7 ± 0.4	13.9 ± 0.5	15.2 ± 0.6
30–45	0.2 ± 0.002	0.3 ± 0.004	8.7 ± 0.2	8.7 ± 0.2	8.9 ± 0.4	6.9 ± 0.3
45–60	0.2 ± 0.001	0.2 ± 0.003	7.1 ± 0.1	6.4 ± 0.1	6.4 ± 0.4	4.1 ± 0.1
September 2019						
0–15	0.3 ± 0.002	0.3 ± 0.006	45.8 ± 0.7	109.2 ± 2.5	23.0 ± 0.6	74.6 ± 2.6
15–30	0.3 ± 0.001	0.3 ± 0.006	19.9 ± 0.6	44.2 ± 0.9	17.5 ± 0.7	34.7 ± 1.1
30–45	0.3 ± 0.002	0.3 ± 0.009	9.6 ± 0.2	34.3 ± 1.0	11.2 ± 0.6	29.5 ± 0.8
45–60	0.3 ± 0.002	0.3 ± 0.012	5.9 ± 0.1	18.5 ± 0.7	8.6 ± 0.5	15.7 ± 0.6

^1^ Soil-2× soil applied UF/IFAS recommendations (2× = 2.24 kg B ha^−1^; 20.16 kg Mn ha^−1^; 11.20 kg Zn ha^−1^).

**Table 5:** Pearson’s correlation coefficient (r) comparing soil Mn with B, Zn, Fe, and Cu as a function of soil Mn application for HLB-affected 2-year-old ‘Valencia’ (*Citrus sinensis* (L.) Osbeck) trees. Total sample (N = 12) for each category.

Element	r	*p*-Value
Boron	−0.76	0.0045
Zinc	−0.69	0.0127
Iron	0.49	0.1041
Copper	0.65	0.0215

## Data Availability

Not applicable.
